# Xenobiotics—Division and Methods of Detection: A Review

**DOI:** 10.3390/jox11040009

**Published:** 2021-10-26

**Authors:** Tea Štefanac, Dijana Grgas, Tibela Landeka Dragičević

**Affiliations:** Faculty of Food Technology and Biotechnology, University of Zagreb, Pierottijeva 6, 10 000 Zagreb, Croatia; tstefanac@pbf.hr (T.Š.); dgrgas@pbf.hr (D.G.)

**Keywords:** xenobiotics, division, detection methods

## Abstract

Xenobiotics are compounds of synthetic origin, usually used for domestic, agricultural, and industrial purposes; in the environment, they are present in micropollutant concentrations and high concentrations (using ng/L to µg/L units). Xenobiotics can be categorized according to different criteria, including their nature, uses, physical state, and pathophysiological effects. Their impacts on humans and the environment are non-negligible. Prolonged exposure to even low concentrations may have toxic, mutagenic, or teratogenic effects. Wastewater treatment plants that are ineffective at minimizing the release of xenobiotic compounds are one of the main sources of xenobiotics in the environment (e.g., xenobiotic compounds reach the environment, affecting both humans and animals). In order to minimize the negative impacts, various laws and regulations have been adopted in the EU and across the globe, with an emphasis on xenobiotics removal from the environment, in a way that is economically, environmentally, and socially acceptable, and will not result in their accumulation, or creation of compounds that are more harmful. Detection methods allow detecting even small concentrations of xenobiotics in samples, but the problem is the diversity and mix of compounds present in the environment, in which it is not known what their effects are). In this review, the division of xenobiotics and their detection methods will be presented.

## 1. Introduction

Urbanization, population growth, industrialization, and globalization are affecting our lives in both positive and negative ways, but they are definitely bringing about change [1]. The connections between countries, technological progress, and the expansion of the market (and the world as a global village) contribute toward global problems, such as the centralization of economies and the facilitation and simplification regarding the movement of goods and services. Nevertheless, despite the benefits, from an economic and political point of view, the impact of globalization on the environment is negative, and a quality environment is a prerequisite for quality of life [2]. Technological progress, longer life, better access to medicine (for humans and animals), as well as daily use of personal care products and/or pesticides, introduce new substances into the environment [3,4]. These substances can cause problems and should be studied in detail, including their short- and long-term effects on humans, animals, and the ecosystem (air, water, and soil), either as single substances or as a mixture of them. Insufficient education on the proper disposal and use of drugs, as well as the lack of interest from some companies in the environment, have led to environmental problems, although awareness of the problem is improving every year [5]. Pollution occurs because manmade contaminants cannot degrade naturally in the environment (or it takes a long time), and science has not found a sufficiently good or applicable solution for artificial degradation that would satisfy all conditions [6]. The term xenobiotics comes from the Greek xenos (foreign) and bios (life), meaning foreign things in living form. The occurrence of xenobiotics in freshwater can, in large part, be attributed to wastewater systems and wet weather run-off [7,8,9].

Wastewater treatment plants are often not effective enough at removing xenobiotics from wastewater, causing xenobiotics to enter public sewers and into the food chain, directly affecting humans [7,10,11] and contributing toward polluting water bodies with micropollutants [12,13,14]. Although communities of bacteria and other microorganisms are shown to be effective at degrading individual xenobiotics, activated sludge is often not specific enough for this task. Communities would have to adapt to the operating conditions and xenobiotics in wastewater [15,16], which are economically unviable in conventional plants. Biological or physicochemical processes that are more effective at removing xenobiotics from water are being extensively researched and improved, which will be discussed later. Removal efficiencies of certain xenobiotics, such as pharmaceutical and personal care products (PPCPs), were found to be highly dependent on technology, and secondary treatment processes were found to be variable (and not fully effective) at removing pharmaceutical contaminants [17].

To prevent or minimize the negative effects of xenobiotics, and to list the priority pollutants, numerous European and worldwide organizations, such as the European Environment Agency (EEA), the United States Environmental Protection Agency (US EPA), and the European Medicine Agency (EMA) have conducted research on their impacts on the environment, humans, animals, contaminant concentrations present in the environment, and the methods to detect harmful compounds. The US EPA defines xenobiotics as new, having environmental and health effects, and as being poorly understood in terms of damage. Various directives and regulations aim to improve quality of the environment by regularly monitoring a list of hazardous compounds. For example, Directive 2013/39/EU of the European parliament and of the council amending Directives 2000/60/EC (Water Framework Directive (WFD)) and 2008/105/EC, in regard to priority substances in the field of water policy, its main directive about pollution of surface water (found at https://eur-lex.europa.eu/homepage.html, accessed on 5 June 2021) [18]. This shows that, as a matter of priority, the causes of pollution should be identified and pollutant emissions should be dealt with at the source, in the most economically and environmentally effective manner. Directive 2000/60/EC [18] established the first list of 33 priority substances or groups of substances in the field of water policy, and Directive 2008/105/EC lays down environmental quality standards (EQS) for those priority substances. The list of priority substances is constantly updating according to high-quality monitoring data and data on ecotoxicological and toxicological effects. Directive 2013/60/EC contains a list of 45 priority substances in the field of water policy. The EQS for those substances contain the latest available scientific and technical information. One of the reports on the modes of action (MoA), and the effects of priority substances and other substances in the Watch List (WL) in the WFD, is in the “Modes of action of the current Priority Substances list under the Water Framework Directive and other substances of interest.“ In that report, information about analyzing these substances by effect-based methods (biomarkers and bioassays), with emphasis on mixtures of substances and their potential interactions in the aquatic environment, are given. Secondly, they group priority substances into 17 groups and watch list substances into 8 groups. The European Medicine Agency offers scientific guidelines on the most appropriate way to fulfill legal obligations applicable to medicinal products in the European Union (https://www.ema.europa.eu/en, accessed on 5 June 2021) [19]. Monitoring of 156 emerging polar organic contaminants in 90 wastewater treatment plant effluents at the European Union level was conducted [20]. Moreover, 80% of the target compounds (125 substances) were found in different concentrations (from nanograms to milligram per liter).

## 2. Xenobiotics

**Figure 1:**
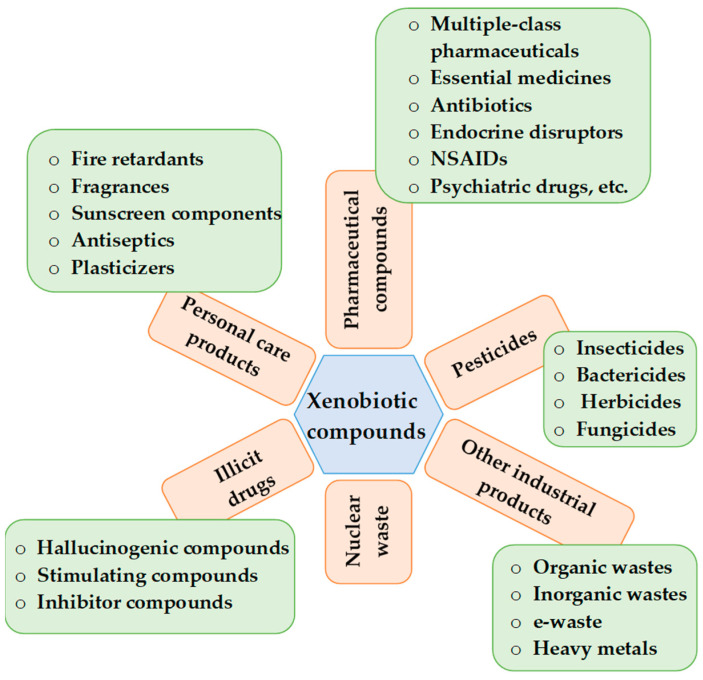
Xenobiotic compounds (modified by [22]).

Technological progress in the 20th century has led to the development of many compounds used to improve daily life (antibiotics, pesticides, dyes, PCPs, additives, etc.) that do not necessarily occur naturally in the environment or whose naturally occurring concentrations are significantly different from those caused by anthropogenic activity. The main problem is their physicochemical structures, such as small molecular size, ionizability, water solubility, lipophilicity, polarity, and volatility [11], which make them difficult to identify, quantify, and remove. Xenobiotics are defined as “chemicals found but not produced in organisms or the environment. Some naturally occurring chemicals (endobiotics) become xenobiotics when present in the environment at excessive concentrations” [21]. They are found in the air, soil, water, plants, animals, and humans, and are classified as pesticides, pharmaceutical compounds, personal care products, illicit drugs, industrial products, and nuclear waste [22], according to Kumar and Chopra, as shown in Figure 1. They are further subdivided (as shown in the same figure). According to WFD, priority substances are divided into 17 groups (herbicides, polyaromatic hydrocarbons, organophosphorus and organochlorine insecticides, chlorinated solvents, aromatic organochlorine compounds, dioxins, PBBs, BDEs, metals, phthalate, anti-fouling biocide, alkylphenols, pyrethroid insecticides, perfluorinated surfactant, benzene, quinoline fungicide, chloroalkanes, and hexabromocyclododecane), watch list substances into 8 groups (hormones, pharmaceuticals, antibiotics, neonicotinoid insecticides, herbicides, carbamate insecticides, antioxidant, and sunscreen agent), and candidate substances, such as pyrethroid insecticides, sulfonylurea herbicide, organophosphorus insecticides, and metals and non-metal trace elements are present. There are various anthropogenic activities for entering xenobiotics into the environment, such as human consumption and excretion, wastewater and sewage treatment plants, livestock treatment and excretion, industries and production plants, and agriculture practices [22]. Pesticides are applied directly to the soil and carried by rain into rivers, groundwater, etc. PPCPs are consumed by humans and enter the environment indirectly, as they cannot be completely metabolized, but are only converted into various metabolites, some of which are more toxic than the parent molecule. After excretion, they end up in sewage/wastewater treatment plants and eventually in rivers, lakes, oceans, soil, groundwater, etc. Both pesticides and PPCPs are taken up by plants and aquatic organisms and enter the food chain. Classification of xenobiotic substances and sources can be made (as in Table 1) [23].

**Figure 2:**
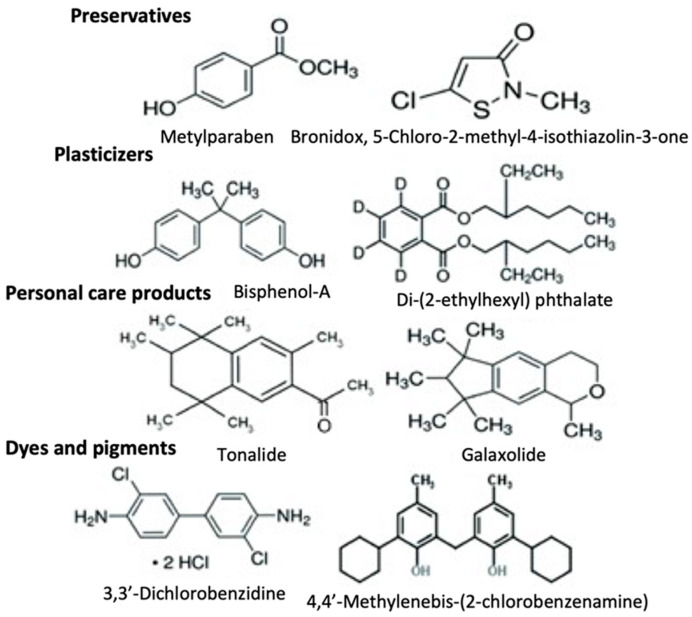
Structure of some xenobiotics (Reproduced with permission from [24]; published by Springer eBook, 2019).

**Figure 3:**
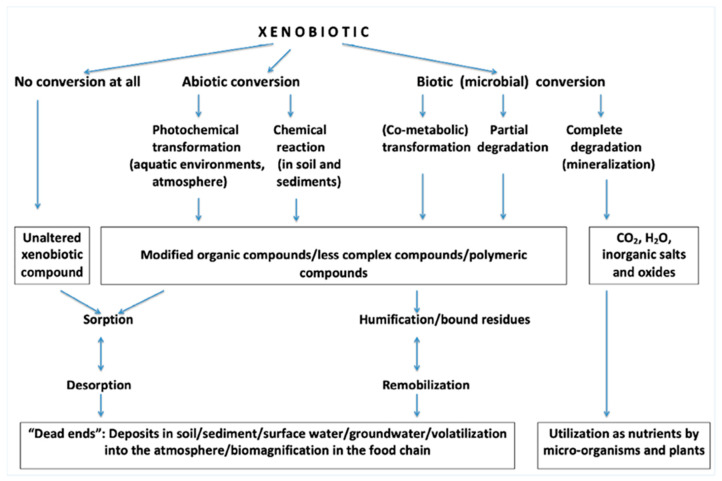
Possible environmental fate of a xenobiotic compound (Reproduced with permission from [5]; published by Springer eBook, 2016).

Xenobiotics are difficult to degrade because of their complex structures, as seen in Figure 2 [24]; thus, they can accumulate in living organisms. Their partial degradation can result in even worse threats. To minimize the concentration of xenobiotics in the environment, it is crucial to know where these compounds are coming from. As shown in Table 1 [23], pollutants can be released directly in the environment or via indirect sources, such as via hospital discharge. Moreover, xenobiotics can be released during a process or as a final product. They can affect the environment deliberately or accidentally, from moving (e.g., a car) or stationary sources (industry). There are many directives and regulations for releasing of xenobiotics into the environment, as mentioned previously, so that sources can be regulated; however, xenobiotics coming from households are difficult to regulate, so it is important to educate people on how xenobiotics are affecting the environment, in the short- and long-term. The possible environmental fate of a xenobiotic is shown in Figure 3 [5]; after all of the steps, they can be deposited/volatilized/biomagnified or mineralized.

Environmental pollutants cause more than 13 million deaths worldwide each year [25]. Their effect on the aquatic ecosystem also causes many problems. Municipal wastewater, along with hospital and manufacturing wastewater, is the largest source of pharmaceuticals [7,26]. Veterinary drugs enter the aquatic environment through surface application, runoff, or direct application [4]. The biofilm community is disrupted by some pharmaceuticals and this affects the entire ecosystem. Effects of xenobiotics on animals are most likely seen on their reproduction and immune functions [27,28]. With the growing influence of herbal medicines worldwide, plants with pharmacological properties should be handled with care, so as not to contaminate crops, vegetables, and surface water. Many pesticides, such as herbicides, fungicides, and rodenticides, are harmful to animals and humans, causing cancer, lung irritation, or neurological disorders [5,29]. The best way to protect the environment and still use pesticides for their beneficial effects on agriculture is to dispose of them properly. The mechanisms by which environmental factors alter basic biological processes to trigger autoimmune diseases continue to be studied, but are still largely unknown [30,31].

## 3. Methods

Determining xenobiotics in environmental samples is challenging because the compounds are often present at low concentration levels that are difficult to detect, and there are a variety of them in different sample types [32,33]. Appropriate extraction and analytical methods for separation and determination of mixtures of xenobiotics and derivatives are important, and they must be rapid, accurate, and at acceptable costs [33,34]. Common sample handling steps in most analytical methods include sample pretreatment, extraction of analytes from the matrix, purification of extracts to remove interferences, and concentrations to achieve the desired sensitivity. In recent years, undeniable progress has been made in the development of techniques to prepare samples for analysis, such as quick, easy, cheap, effective, rugged, and safe (QuEChERS), solid phase extraction (SPE), solid phase microextraction (SPME), stir bar sorptive extraction (SBSE), hallow-fiber liquid phase microextraction (HFLPME), dispersive liquid–liquid microextraction (DLLME), or focused ultrasonic solid-liquid extraction (FUSLE), and others [33]. The QuEChERS method is used in multi-residue pesticide analysis, in analysis of antibiotics, hormones, mycotoxins, polycyclic aromatic hydrocarbons, and persistent organic pollutants, such as dioxins and polychlorinated biphenyls in food and environmental matrices. QuEChERS is usually combined with gas chromatography–mass spectrometry (GC–MS) or liquid chromatography–mass spectrometry (LC–MS) analysis, which give them high selectivity, sensitivity, and specificity [35]. SPE involves preparation techniques for organic contaminants from environmental matrices. It is used for analysis of pesticides, carbamate, bisphenols, phthalate acid esters, and pharmaceuticals [36]. SPME enables sampling and sample preparation simultaneously, and is used for analyzing of pesticides, polycyclic aromatic hydrocarbons, phenols, amines, and polychlorinated bisphenols in food and environmental samples [37]. SBSE is used in environmental analysis, food analysis, and life science and biomedical analysis. In environmental and food analysis, it is used for determination of pesticides, polycyclic aromatic hydrocarbons, phenols, pharmaceuticals, alkylphenols, chlorophenols, bisphenol A, mycotoxins, and fungicides [38]. HFLPME with a porous hollow fiber membrane is used for trace analysis of heavy metals, such as lead or arsenic, pharmaceuticals, and other organic compounds in environmental, clinical, and biological samples, petroleum products, pharmaceuticals, and in food. It is compatible with most analytical instruments for chromatography, electrophoresis, molecular and atomic spectrometry, and electrochemistry [39]. DLLME is applied for organic compounds, such as phthalate esters or parabens, and metal ions, such as cadmium, selenium, and lead, mostly from water samples. The main use is in the analysis of pesticide in the water matrix, and phenols, such as chlorophenols and endocrine disrupting phenols, and pharmaceuticals [40]. FUSLE can be used for determining inorganic, organometallic, and organic compounds, such as polycyclic aromatic hydrocarbons, polychlorinated biphenyls, phthalate esters, and nonylphenols from environmental samples. It can also be used for determining endocrine disrupter compounds (bisphenol A and alkylphenols) in sewage sludge [41,42]. Xenobiotics analysis includes very sensitive and selective analysis techniques,, such as chromatographic methods—high-performance liquid chromatography (HPLC), ultrahigh-performance liquid chromatography (UPLC), gas chromatography (GC), and multidimensional chromatographic techniques, often coupled with modern detection techniques (high resolution mass spectrometry— HRMS) [11]. Chromatographic analysis of xenobiotics are used for separation and determination of compounds with similar chemical structures in the air, ground, in surface water, sludge, soil matrices, food and food products, and in human and veterinary health care. GC methods need compounds that are volatile or semi-volatile, such as toluene, xylene, and acetaldehyde. HPLC is employed for the determination of phenols and polycyclic aromatic hydrocarbons, such as acenaphthene, fluorene, pyrene, chrysene, and fluoranthene in water and soil, and polychlorinated biphenyls [43]. UPLC reduces analysis time and it is used for detection of pharmaceuticals, mycotoxins, and pesticides [44]. HPLC and UPLC are frequently applied with MS or/and MS/MS. Multidimensional chromatography improves the resolution and separation power. With HRMS, an unlimited number of xenobiotics can be simultaneously analyzed because full-scan data are collected, rather than preselected ion transitions corresponding to specific compounds [33]. Another suitable method is the enzyme-linked immunosorbent assay (ELISA), which offers a new approach for the detection of pharmaceutical compounds in surface water [45,46]. ELISA is suitable for the quantitative analysis for chemicals such as bisphenol A, diethylhexyl phthalate, dibutyl phthalate, alkylphenol, alkylphenol ethoxylate, and chlorophenols, pesticides, carbamates, organochlorine, and organophosphorus compounds. It allows the simultaneous analysis of a large number of samples [47]. Sensors are great tools for xenobiotics detection and monitoring, and are composed of nanomaterials, recognition elements, and a signal transduction means for analyte detection. They detect environmental pollutants, such as pesticides, heavy metals, polycyclic aromatic hydrocarbons, toxins, and other emerging contaminants, including gasoline additives, pharmaceuticals, hormones, personal care products, endocrine-disrupting agents, organometallic compounds, disinfection by-products, plasticizers, perfluorinated compounds, and surfactants [48]. They are easy to use, portable, sustainable, and are cost-effective [49]. Some pesticides that can be detected by using biosensors are paraoxon, acetamiprid, atrazine, and fenitrothion. They can also detect metals, such as mercury, lead, chromium, toxins, and endocrine disrupting chemicals. The detection of persistent pollutants and heavy metals in aqueous samples can be performed with sensors and biosensors, such as an AFM tip nanobiosensor with acetyl-CoA carboxylase, an immunosensor based on a modified carbon printed electrode, an electrochemical sensor based on polymeric electrospun nanofibers of polyamide 6 (PA6)/polypyrrole (PPy) surface-modified with two forms of graphene, a biosensor with double encapsulated algae strains *Chlorella vulgaris* and *Pseudokirchneriella subcapitata* alginate beads/silica gel, a biosensor containing recombinant *Escherichia coli* [50,51,52,53,54]. They can convert the information about the presence of the pollutant into a measurable signal; if it is a biosensor, the element used to detect the analyte is biological.

**Figure 4:**
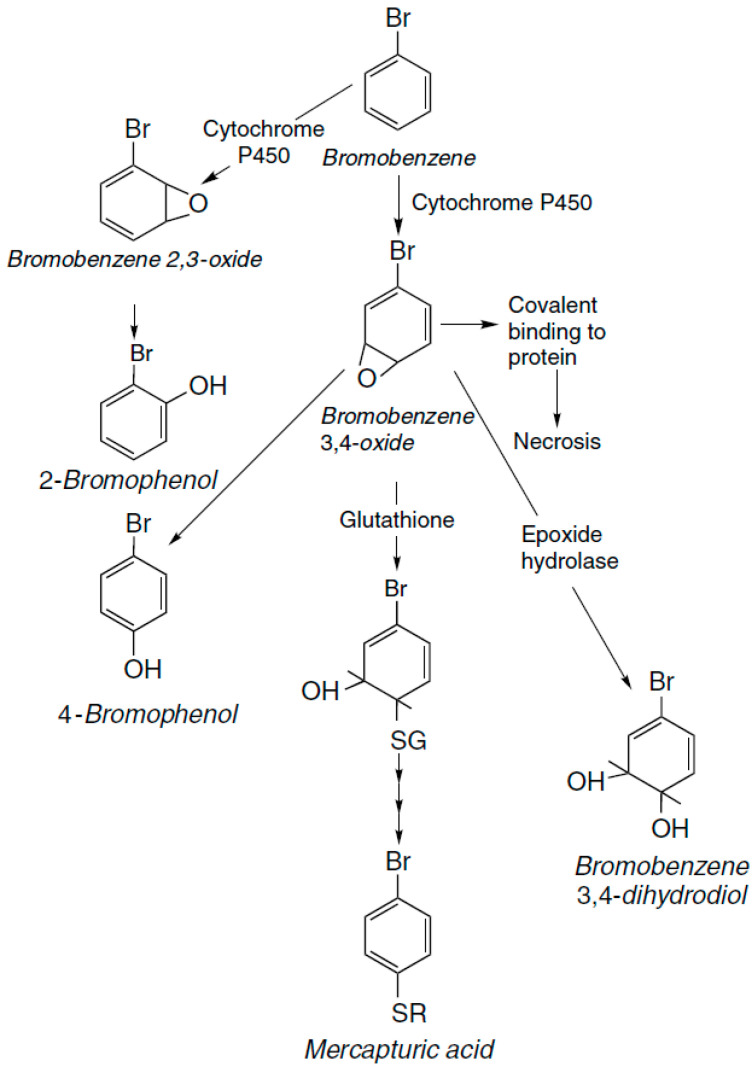
Metabolic pathways for bromobenzene (Reproduced with permission from [63]; published by Wiley, 2011).

Xenobiotics in wastewater are a major problem, as mentioned earlier. There are several methods that are suitable for the removal of xenobiotics, such as biotransformation, degradation, adsorption, advanced oxidation processes, constructed wetlands, and membrane processes [7,55,56,57,58,59,60,61,62]. Biotransformation or bioconversion is a process of converting compounds from one form to another, which is easily excreted. It is divided into three phases—functionalization, conjugation, and elimination [23]. Biotransformation is almost always catalyzed by enzymes. Example of biotransformation on paracetamol includes three pathways of metabolism—sulfate, glucuronic acid conjugation, and conjugation with glutathione. The last pathway involves a reaction catalyzed by cytochrome P450, producing a reactive metabolite, which can be detoxified by conjugation with glutathione and then metabolized to a cysteine conjugate, which is acetylated and excreted as an *N*-acetylcysteine conjugate or mercapturic acid. Bromobenzene is a hepatotoxin and is metabolically activated by oxidation, catalyzed by cytochrome P450. Intermediate 3,4-epoxide can be detoxified by conjugation with glutathione, giving mercapturic acid conjugate, which is excreted in the urine. Another pathway catalyzed by P450 gives rise to the 2,3-epoxide. The third way of detoxification is metabolism to the dihydrodiol mediated by epoxide hydrolase. Some of the metabolic pathways for bromobenzene are given in Figure 4. Methanol is toxic, mainly as a result of metabolism to formic acid in a two-step reaction, with formaldehyde as a first metabolite. The first step is catalyzed by alcohol dehydrogenase or catalase, and second by aldehyde dehydrogenase or formaldehyde dehydrogenase [63]. Degradation techniques are classified as bioremediation (microbial remediation with bacteria, fungi, and algae, and phytoremediation) and photoremediation. In photoremediation UV, IR, and visible radiation from the sun are used to degrade xenobiotic compounds, such as pesticides, heavy metals, and dyes, which have the ability to adsorb photons. Due to development of photodegradable polymer, photoremediation can be used for the degradation of plastic. Advances to degrade Congo red dye include photocatalytic degradation using ZnO/UV-A. Photodegraded films are used to evaluate biodegradation using microorganisms, such as *Aspergillus niger* and *Penicillium funiculosum* for degradation of both natural and synthetic plastics [64]. Sertraline is a drug that can be found in surface waters and its main transformation pathway is phototransformation. It is degraded dominated by direct photolysis and the reactive species further accelerate the compound’s breakdown rite [65]. Bioremediation uses microorganisms or plants (and/or their metabolites) to reduce the concentration of a pollutant (metals, minerals, nitrogen, sulfur, etc.) in the environment under specific environmental factors (temperature, pH). Bioremediation can be achieved in two ways: in situ (direct approach at the site of pollution) and ex situ (on designated place). There are about 50 microbial strains that have been isolated and that have the capacity to degrade xenobiotics [66]. Moreover, with the use of GMOs in bioremediation, some limitations of this method can be overcome. Phytoremediation uses living plants, such as *Carex pendula*, *Elodea canadensis*, *Juncus articulates*, or *Vallisneria spiralis* [23] for remediation of sludge, soil, ground water, and sediments. Enzymes, such as oxidoreductases and hydrolases–monooxygenases, dioxygenases, peroxidases, and laccases, are important for degradation of xenobiotic compounds (heavy metals, aromatic compounds, petroleum derivatives, dyes, estrogenic substances, phenols, polyamines etc.). They are more advantageous compared to microorganisms themselves because of greater process control, faster action, and more efficient treatment [55,57]. Bacteria, such as *Pseudomonas, Gordonia*, *Bacillus*, *Moraxella*, *Micrococcus*, *Escherichia*, *Sphingobium*, *Pandoraea*, *Rhodococcus,* can be used for degradation of petroleum hydrocarbons (*Bacillus subtilis*, *Pseudomonas putida*, *P. aeruginosa*, *Micrococcus* sp.), pesticide glyphosate (*P. putida*, *Acinetobacter faecalis*), tetrachlorvinphos (*Vibrio metschnikovii*, *Proteus vulgaris*), atrazine (*Enterobacter* spp., *Bacillus* spp.), and organochlorine (Actinomycetes). For removing PCP (pentachlorophenol) from contaminated water, bioreactors with alginate immobilized along with Polyurethane foam immobilized PCP degrading Flavobacterium cells have been used [67]. Members of the *Alicycliphilus* genus are environmental bacteria, which have the ability to use oxygen, nitrate, and chlorate as electron acceptors. That allows them to degrade xenobiotics under oxic or anoxic conditions. They can biodegrade acetone, cyclohexanol, N- methylpyrrolidone, benzene, toluene, polyurethane varnishes, triclosan, and antibiotics. Biodegradation of acetone with *Alicycliphilus* sp. is carried out by carboxylation of acetone to acetoacetate, catalyzed by acetone carboxylase, activation to acetoacetyl-CoA and cleaving into two acetyl-CoA molecules [68]. Treatment with activated carbon removes compounds by physical adsorption onto activated carbon (AC) bed, which needs to be replaced/regenerated after some time. It is one of the most used technologies, and it can remove up to 90% of xenobiotics [7]. However, AC is expensive and there is a need for identifying alternative adsorbent materials for affordable and efficient xenobiotic removal [61]. Advanced oxidation processes, such as Fenton, photolysis process, or ozonization have high efficiency in treating organic compounds. They use strong hydroxyl or sulfate radicals as the main oxidants that can easily break down pollutants and remove them. Sometimes target substance do not degrade completely, and sub-products can present higher toxicity that the original compound, so the toxicity evaluation of the sub-products are necessary [56,58,62]. Filtration methods, such as nanofiltration and reverse osmosis, use membranes with different pore sizes as physical barriers to remove compounds from the effluent of wastewater, with several mechanisms, such as steric exclusion, adsorption, diffusion, and electrostatic interactions. Membrane filtration for the removal of xenobiotics is a physical process and it does not produce unwanted sub-products. The removal mechanisms for each process are determined by the characteristics of the xenobiotics, the membrane type, the water matrix and solution chemistry, and the operating parameters [69,70]. Constructed wetlands (CWs) can be defined as designed structures consisting of waterlogged beds planted with emergent and/or submerged vegetation. They simulate natural wetlands and include physical, chemical, and biological processes. CW technology is used in pharmaceuticals, pesticides, dyes, explosives, hormones, and PCP removal, among others [59]. The support matrix is a critical component of CWs, the careful selection of which can lead to significant increases in the efficiencies of these systems. Thus, due to its importance, the composition of the support matrix is a primary issue in CW optimization [60].

## 4. Conclusions

This review provides information on the legislation and classification of xenobiotics, the effect of xenobiotics on the environment, humans, and animals, and how to minimize these effects. It also provides an overview of the method of detection and removal of xenobiotics. The possible environmental fate of xenobiotics is also discussed. Methods for determining xenobiotics, such as QuEChERS, SPE, SPME, SBSE, HFLPME, DLLME, and FUSLE, are listed. Moreover, xenobiotics analysis includes very sensitive and selective techniques, such as HPLC, UPLC, GC, and multidimensional chromatographic techniques, often coupled with modern detection techniques (high resolution mass spectrometry—HRMS). Furthermore, suitable methods for xenobiotic detection include the enzyme-linked immunosorbent assay (ELISA), sensors, and biosensors—they are great tools for xenobiotics detection and monitoring, are easy to use, portable, sustainable, and cost-effective. Xenobiotics are difficult to degrade because of their complex structures and possible accumulation/magnification in living organisms. Partial degradation can result in more harmful compounds than parental molecules. Xenobiotic removal methods, such as biotransformation, bioremediation, photoremediation, adsorption, advanced oxidation processes, constructed wetlands, and membrane processes, are highlighted. In order to minimize the negative effects of xenobiotics and reduce their use, organizations worldwide have passed directives and regulations to monitor them. The list of priority contaminants and harmful compounds is regularly updated with all compounds detected, with the development and modernization of methods.

## Figures and Tables

**Table 1:** Substances and sources of xenobiotics (modified by [23]).

Classification			
Xenobiotic Substances			Xenobiotic Sources
**characteristics**	**classification**	**example**	**Direct sources**: pharma industries (phenols), petroleum effluent (hydrocarbons), plastics, paints, dyes, pesticides, insecticides, paper and pulp effluent **Indirect sources**: hospital discharge, pesticides or herbicide residues **Product and processes**: product of reaction of any processes–domestic or industrial scale **Deliberate and accidental causes**: chemicals used in paper and pulp industries; released into the environment due to accidents **Moving and stationary**: cars and industries **Regulated and unregulated**: large industries and automobiles household activity
**nature**	Natural	Bacteriotoxins, zootoxins, phytotoxins, serotonin	
	Synthetic	Man-made substances, pesticides	
**uses**	Active	Pesticides, dyes, paints	
	Passive	Additives, carrier molecules	
**physical state**	Gaseous	Benzene, aerosol form	
	Dust-form	Asbestos powder	
	Liquid	Chemicals dissolved in water	
**pathophysiological effects**	Tissue/organs	Kidney toxins	
	Biochemical mechanism	Methemoglobin producing toxins	
